# Potassium Ion Channel Gene *OsAKT1* Affects Iron Translocation in Rice Plants Exposed to Iron Toxicity

**DOI:** 10.3389/fpls.2019.00579

**Published:** 2019-05-08

**Authors:** Lin-Bo Wu, Felix Holtkamp, Andriele Wairich, Michael Frei

**Affiliations:** ^1^Department of Plant Nutrition, Institute of Crop Science and Resource Conservation, University of Bonn, Bonn, Germany; ^2^Center for Biotechnology, Federal University of Rio Grande do Sul (UFRGS), Porto Alegre, Brazil

**Keywords:** abiotic stress, iron toxicity, potassium, potassium ion channel, rice, tolerance

## Abstract

Iron toxicity is one of the most widely spread mineral disorders in anaerobic soils, but the tolerance mechanisms in plants are poorly understood. Here we characterize the involvement of a rice potassium ion channel gene, *OsAKT1*, in Fe toxic conditions. Two knock-down lines of *OsAKT1* together with azygos lines were investigated. Mutant lines did not differ from azygos lines regarding plant growth, gas exchange rate or chlorophyll fluorescence in control conditions. However, loss-of-function of *OsAKT1* increased the sensitivity to excess Fe regarding leaf bronzing symptoms, reactive oxygen species generation, leaf spectral reflectance indices, and chlorophyll fluorescence. Fe toxicity leads to largely reduced uptake of other nutrients into shoots, which illustrates the complexity of Fe stress related to multiple mineral disorders. Less potassium uptake in the mutants compared to azygos lines co-occurred with higher amounts of Fe accumulated in the shoot tissues but not in the roots. These results were consistent with a higher level of Fe loaded into the xylem sap of mutants compared to azygos lines in the early phase of Fe toxicity. In conclusion, *OsAKT1* is crucial for the tolerance of rice against Fe toxicity as K homeostasis affects Fe translocation from root to shoot.

## Introduction

Iron (Fe) is a crucial mineral element for all living organisms due to its property as an electron receptor/donor (Kobayashi et al., 2013). Despite its various roles in photosynthesis, respiration and other physiological processes in plants, Fe is toxic when presenting in excess (Becker and Asch, 2005). In optimal growth conditions, rice plants employ both reduction-based (Strategy I) and chelation-based (Strategy II) strategies to take up Fe from the soil (Römheld and Marschner, 1986; Bughio et al., 2002; Ishimaru et al., 2006). In lowland rice fields that contribute 95% of the world rice production, Fe prevalently occurs in the reduced and soluble ferrous form (Fe^2+^) due to the low soil redox potential arising from anaerobic conditions, which are formed when soil microorganisms and plant roots deplete oxygen by respiration. Excessive Fe^2+^ are transported via the xylem flow to the shoot leading to Fe toxicity, which is one of the most widely observed nutrient disorders in lowland rice production (Becker and Asch, 2005; Frei et al., 2016). Around 12% of the rice production area in Africa is potentially affected by Fe toxicity (van Oort, 2018). Once excess Fe^2+^ enters the plant cells, they can participate in Fenton reactions, in which Fe^2+^ reacts with hydrogen peroxide (H_2_O_2_) leading to hydroxyl (OH⋅) radical production. At the same time, Fe^2+^ is oxidized to Fe^3+^, which can be reduced back to Fe^2+^ by reducing agents such as ascorbate (AsA) (Grillet et al., 2014; Wu et al., 2017). Hydroxyl radicals can irreversibly damage various cell components such as DNA, proteins, and lipids (Becana et al., 1998) leading to the formation of visible bronzing spots on leaves (Ponnamperuma et al., 1955). Moreover, Fe toxicity can limit the photosynthesis rate through damaging chlorophyll molecules (Stein et al., 2014). High amounts of free Fe^2+^ in soil solutions can directly damage the root system leading to the reduced uptake of essential nutrients (Wu, 2016). Eventually, rice yield production under Fe toxic conditions is negatively affected. Depending on the timing of occurrence and stress density, the yield loss can range from 15 to 100% (Audebert and Fofana, 2009).

To cope with Fe toxicity, rice plants evolve different tolerance mechanisms, which can be classified into two major categories: exclusion and inclusion. A well-characterized exclusion mechanism is related to the oxidation and precipitation of Fe at the root surface, where root plaque is formed to prevent further excess Fe uptake. This process is mediated by root oxidizing power favored by root architectural traits, which facilitate the transport of oxygen from shoot to root and the diffusion into rhizosphere (Wu et al., 2014). Inclusion mechanisms are associated with the partitioning of excess Fe in the tissues with lower photosynthetic activities such as the leaf sheath (Engel et al., 2012). Vacuole constitutes the main organelle for storing excess free Fe in plants (Thomine and Vert, 2013). Moreover, the ubiquitous Fe-storage protein Ferritin, which can store up to 4000 Fe atoms, was shown to be involved in the tolerance to Fe toxicity in rice (da Silveira et al., 2009; Stein et al., 2009). Additionally, rice plants can achieve tolerance to Fe toxicity through maintaining low redox potential of AsA to avoid the pro-oxidant activity of reduced AsA, because AsA can directly reduce Fe^3+^ to Fe^2+^ thus stimulating the Fenton reaction leading to further oxidative stress (Wu et al., 2017).

Genetic factors of Fe toxicity tolerance in rice have been investigated in many studies using either bi-parental populations or diversity panels (Dufey et al., 2014; Wu et al., 2014; Matthus et al., 2015; Zhang et al., 2017). No major quantitative trait loci (QTL) but rather small to medium effect multi-loci have been mapped so far, pointing to a complex genetic architecture of Fe tolerance in rice. In our previous genome-wide association study (GWAS), we suggested a potassium ion channel gene (*OsAKT1*, LOC_Os01g45990) to be associated with the regulation of shoot Fe concentration (Matthus et al., 2015). *OsAKT1* belongs to the Shaker gene family and encodes an inward potassium ion (K^+^) channel that localizes on the plasma membrane (Li et al., 2014). *OsAKT1* acts as one of the essential K^+^ channels mediating the K uptake in rice (Fuchs et al., 2005; Obata et al., 2007). K constitutes the most abundant monovalent cation in plant cells and plays crucial roles in various plant physiological processes including photosynthesis, assimilation products transport, and resistance/tolerance to biotic/abiotic stresses (Wang and Wu, 2013). Maintaining adequate K status is essential for plants to confer tolerance to drought (Cakmak, 2005), salinity (Garthwaite et al., 2005), and submergence (Gautam et al., 2016). Under excess Fe conditions, higher sensitivity in *Arabidopsis* plants was associated with impaired K homeostasis due to the NO-mediated K loss possibly via non-selective cation channels (NSCC) (Zhang et al., 2018). In rice, interactions between K status and Fe uptake under excess Fe conditions were observed in several studies (Li et al., 2001; Gao et al., 2014) but the underlying genetic and physiological mechanisms, as well as the role of endogenous K homeostasis in Fe toxicity tolerance, remain elusive.

The aims of this study were (i) to confirm the involvement of the *OsAKT1* gene in Fe toxicity tolerance as proposed in our previous study (Matthus et al., 2015). To achieve this goal, two independent rice mutants of *OsAKT1* were investigated in Fe toxic conditions (1,000 mg L^-1^ = 17.9 mM Fe^2+^ for 5 days) in hydroponics. Plant responses including stress symptoms, reactive oxygen species (ROS) generation, leaf spectral reflectance, gas exchange, and photosynthetic activities were examined in mutants and azygos lines. (ii) Further, we aimed to unravel mechanisms by which K homeostasis affects Fe toxicity tolerance, and the mineral composition of Fe stressed rice plants. To this end, ionomic profiling of different plant tissues along with physiological experiments was conducted with mutant lines for the *OsAKT1* gene.

## Materials and Methods

### Plant Materials

Seeds of two lines NG1928, and NC2778 in Nipponbare (*Oryza sativa* L., ssp. *japonica*) background carrying retrotransposon Tos17 insertions in *OsAKT1* (Potassium ion channel, LOC_Os01g45990) were obtained from the Rice Genome Resource Center of the National Institute of Agrobiological Sciences (NIAS), Tsukuba, Japan (Miyao et al., 2003). According to the Tos17 database^1^, gene-specific and Tos17-tail6 primers (Supplementary Table S1) were used to genotype the mutant lines. Plants carrying homozygous insertions together with co-segregating wildtypes (azygos) were grown to maturity to obtain seeds for further experiments.

### Plant Culture and Screening Experiment

Rice seeds were first soaked in de-mineralized water and germinated for 3 days in the dark at 30°C. Young seedlings were subsequently floated on the solutions containing 0.5 mM CaCl_2_ and 10 μM FeCl_3_ in the light for another 7 days in a climate-controlled glasshouse. Natural light was supplemented with artificial lighting to ensure a minimum photosynthetic photon flux density (PPFD) of 600 μmol m^-2^ s^-1^. The day/night temperature was set to 28/22°C. Homogenous seedlings were selected and transplanted into the 60-L tanks filled with half-strength Yoshida solution with modification (Yoshida et al., 1976; Shrestha et al., 2018). One week later, nutrient solutions were exchanged to full strength solutions with the following composition: 2.86 mM N (as NH_4_NO_3_), 0.32 mM P (as NaH_2_PO_4_⋅2H_2_O), 1.02 mM K (as K_2_SO_4_), 1 mM Ca (as CaCl_2_), 1.65 mM Mg (as MgSO_4_⋅7H_2_O), 9.1 μM Mn (as MnCl_2_⋅4H_2_O), 0.52 μM Mo (as (NH_4_)_6_⋅Mo_7_O_24_⋅4H_2_O), 18.5 μM B (as H_3_BO_3_), 0.15 μM Zn (as ZnSO_4_⋅7H_2_O), 0.15 μM Cu (as CuSO_4_⋅5H_2_O), 35.7 μM Fe (as Fe-EDTA). The pH value was adjusted to 5.5 every 2 days, and nutrient solutions were renewed every 10 days. To avoid tangling of roots from different plants affecting root oxidizing power, we used PVC tubes fixed underneath a perforated covering plate to create an independent rhizosphere for every single plant grown in the same tank (Wu et al., 2014). After 4-week-growth, FeSO_4_⋅7H_2_O was added to the nutrient solutions to start an acute Fe stress of 17.9 mM Fe^2+^. To prevent the auto-oxidation of Fe^2+^, nitrogen gas was percolated through the nutrient solutions for 15 min every 2 h to remove the dissolved oxygen partially. After five-day-treatment, leaf bronzing symptoms were scaled from 0 (no symptoms) to 10 (dead leaf) according to Wu et al. (2014). Shoot and root tissues were separately flash frozen with liquid nitrogen and stored at -80°C for further analysis. To investigate the distribution pattern of different elements in various tissues, roots, leaf blades, combined leaf sheaths and culms (termed as SC) were harvested separately for mineral analysis. The experiment was designed as a full factorial design with four replicates and six sub-replicates of each genotype per tank.

### Leaf Spectral Reflectance Measurement

Leaf spectral reflectance was monitored on the first and second fully expanded leaves on the main tiller with a PolyPen RP410 device (PSI, Drasov, Czechia) to reveal the stress responses and pigment composition of different lines. Different indices were calculated as follows:

Photochemical reflectance index (PRI) = (R531 – R570)/(R531 + R570) (Gamon et al., 1997); Normalized pigment chlorophyll index (NPCI) = (R680 – R430)/(R680 + R430) (Peñuelas et al., 1994); Greenness index (GI) = R554/R677; Normalized difference vegetation index (NDVI) = (R_NIR_ – R_RED_)/(R_NIR_ + R_RED_) (Huang et al., 2013). In all equations, R represents the reflectance at a given wavelength.

### Gas Exchange and Chlorophyll Fluorescence Measurement

Gas exchange and chlorophyll fluorescence were simultaneously measured with a portable photosynthetic gas exchange system Li-Cor 6400 (LI-COR, Inc., Lincoln, NE, United States). The first and second fully expanded leaves on the main tiller of each plant were measured between 10.00 to 14.00 h on the fifth day after starting the treatment. Reference CO_2_ concentration and PPFD were set to 400 ppm and 900 μmol m^-2^ s^-1^, respectively. Chlorophyll fluorescence parameters were taken after saturating flashes with a ratio of 10:90 of blue: red light once the raw fluorescence value was stabilized. The calculations of different parameters follow Höller et al. (2015). Quantum efficiency of photosystem II (PSII) was calculated as ΦPSII = (Fm’ - Ft)/Fm’ where Fm’ is the maximal fluorescence under actinic light and Ft is the steady-state terminal fluorescence. Other parameters including stomatal conductance, transpiration rate, leaf to air vapor pressure deficit, leaf temperature and net photosynthetic rate were also monitored.

### DNA, RNA Extraction and Quantitative Reverse Transcription PCR

Genomic DNA was isolated using the PeqGOLD plant DNA mini kit (PeqLab Biotechnologie GmbH, Erlangen, Germany) according to manufacturers’ instructions. Total RNA from shoot and root were separately extracted with RNA extraction kits (for shoot samples, peqGOLD RNA kit, Peqlab; for root samples, RNeasy Plant Mini Kit, Qiagen GmbH, Düsseldorf, Germany) following the manufacturers’ instructions. During the extraction procedure, genomic DNA was removed with on-column RNAase-free DNAase (shoot, Peqlab; root, Qiagen). Three replicates of root/shoot RNA from each sample were tested for integrity on a bleach agarose gel (Aranda et al., 2012) and for purity using NanoDrop OneC (Thermo Fisher Scientific, Braunschweig, Germany). Afterward, 300 ng of total RNA was reverse transcribed to cDNA with GoScript Reverse Transcription Kit (Promega, Manheim, Germany). Quantitative RT-PCR was conducted using GoTaq qPCR master mix (Promega) with a Bio-rad CFX384 real-time system (Biorad, Munich, Germany). The reaction conditions were set up as follows: an initial denaturation step (5 min, 95°C) followed by 40 cycles of denaturation (15 s, 95°C), annealing/extension (1 min, 60°C). Relative expression of *OsAKT1* was calculated using the comparative ΔΔCT method with the expression level of azygos lines in the control as calibrator and *OsUBQ5* as the endogenous reference (Ashrafuzzaman et al., 2018). The primer sequences used in this study are listed in Supplementary Table S1.

### H_2_O_2_ Detection in Leaves

*In situ* detection of H_2_O_2_ in leaves was conducted according to Wu et al. (2017). In brief, the first and second youngest fully expanded leaves were detached from the main tiller and washed with 0.05% Triton X-100 followed by rinsing with distilled water three times. Then the leaves were submerged into a solution (pH 3.8) containing 0.5 mg mL^-1^ 3,3′-Diaminobenzidine tetrahydrochloride (Merck KGaA, Darmstadt, Germany) dissolved in Milli-Q water (Merck KGaA) for 12 h in the dark and afterward rinsed again with distilled water. The leaves were subsequently incubated in a bleaching solution containing glycerol, lactic acid, and ethanol at a ratio of 1:1:4 (v/v), at 85°C for 1 h to remove the chlorophyll. H_2_O_2_ formation was visualized as brown precipitation documented by a camera (550D, Canon Deutschland GmbH, Krefeld, Germany).

### Ionomic Profiling in Different Tissues

Rice roots were detached and immersed in the dithionite-citrate-bicarbonate (DCB) solution followed by 4-time rinsing with distilled water to remove the root plaque (Liu et al., 2004). Leaf blades were separated from the shoots leaving the combined leaf sheath and culm. Different tissues including the leaf blade, SC and washed roots were dried at 60°C for 4 days until constant weight. Dry samples were ground to fine powder followed by digestion with 65% HNO_3_ in a microwave pressure digestion system (MARS6, CEM GmbH, Kamp-Lintfort, Germany). The digested samples were then diluted to a final volume of 20 mL. Different elements including Fe, Ca, Mg, Cu, Zn, Mn, Ni, and Mo were measured with an Inductively Coupled Plasma Atomic Emission Spectrometry (ICP-OES, Ultima2, Horiba Jobin Yvon GmbH, Bensheim, Germany). K and Na were measured by Atomic Absorption Spectrometry (AAS, Perkin-ELMER 1100B, Überlingen, Germany). Fe concentration in the root plaque extract was also measured with AAS. Root oxidizing power was calculated as the amount of Fe precipitated on the root surface per root dry weight.

### Xylem Sap Collection and Fe Determination

Xylem sap was collected from the plants grown in both control and Fe treatment. Two hours after starting the Fe treatments, rice shoots were removed at the position of 1 cm above the basal node. Xylem sap was collected for 3 h and transferred into 1.5 mL centrifuge tubes kept on ice. The xylem sap was then incubated in 10 mM ascorbic acid solution to reduce Fe^3+^ to Fe^2+^. The total Fe was measured with the colorimetric method using 2-2′ bipyridyl with a microplate reader (Powerwave XSII, BioTek, Bad Reichenhall, Germany) at the wavelength of 522 nm (Hartmann and Asch, 2018).

### Statistical Analysis

Two-way analysis of variance (ANOVA) was used to analyze the effects of treatment, genotype, and treatment by genotype interaction on different traits with SPSS software (IBM SPSS Statistics 24, Ehningen, Germany). Within treatments, genotypic differences were analyzed with one-way ANOVA at the significance level of *P* < 0.05. *Post hoc* multiple comparisons for observed values were conducted using the Tukey’s test if appropriate. The heatmap for ionomic profiling was generated with Heatmapper^2^ (Babicki et al., 2016). Hierarchical clustering for the variation patterns of different elements was conducted using the average linkage with the Pearson’s test.

## Results

### Genotyping and Identification of Rice Mutants of *OsAKT1*

**FIGURE 1:**
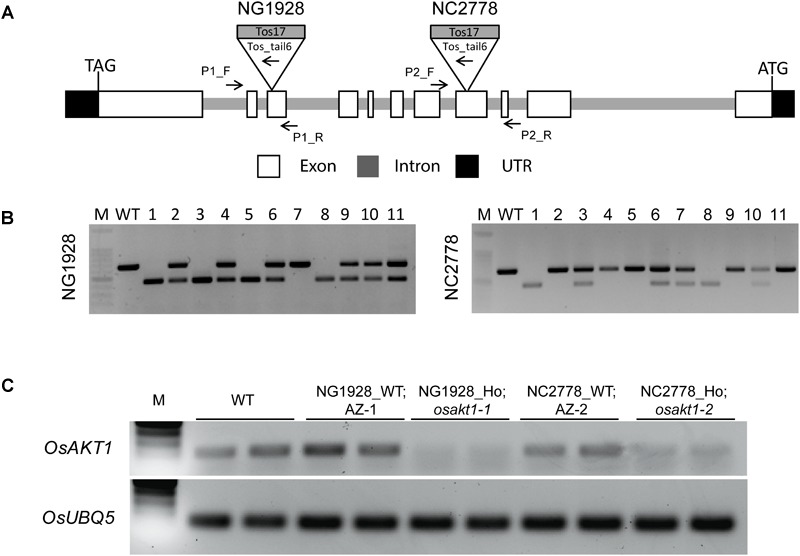
Genotyping and semi-quantitative RT-PCR analysis of the Tos17 insertional lines for potassium ion channel *OsAKT1*. **(A)** Schematic representation of the *OsAKT1* gene structure and Tos17 insertions in two different lines, NG1928 and NC2778. **(B)** Genotyping results of T2 plants of NG1928 and NC2778 line. **(C)** Semi-quantitative RT-PCR of *OsAKT1* in NG1928 and NC2778 segregation lines. Rice ubiquitin gene (*OsUBQ5*) was used as a reference. The sequences of the primers are listed in Supplementary Table S1. P1_F, P1_R: gene-specific primers for NG1928; P2_F, P2_R: gene-specific primers for NC2778; Tos_tail6: Tos17 primer; M: DNA marker; WT: Nipponbare; AZ-1: azygos line of NG1928; AZ-2: azygos line of NC2778.

T2 plants of NG1928 and NC2778 were genotyped using triple primer PCR with gene-specific primers and Tos17-tail6 primer (Figure 1A). Homozygous plants showed single PCR band differing from wildtype (WT) while heterozygous plants showed two bands (Figure 1B). Homozygous lines from NG1928 (plant ID 1, 3, 5 and 8) and NC2778 (Plant ID 1,8) were grown to maturity to obtain T3 seeds together with azygos (AZ) lines (for NG1928, plant ID 7; for NC2778, plant ID 2, 4, 5), in which mutant alleles were segregated out. Semi-quantitative RT-PCR was conducted using homozygous T3 plants (Figure 1C) together with their corresponding AZ lines. The homozygous mutant lines of both NG1928 and NC2778 showed reduced expression compared to AZ lines indicating that insertion of Tos17 led to the knock-down effect of *OsAKT1* gene. The mutant lines from NG1928 and NC2778 were named as *osakt1-1* and *osakt1-2*, respectively. AZ lines from NG1928 and NC2778 were accordingly named as AZ-1 and AZ-2, respectively.

### Responses of *OsAKT1* Mutant Lines to Fe Toxicity

**FIGURE 2:**
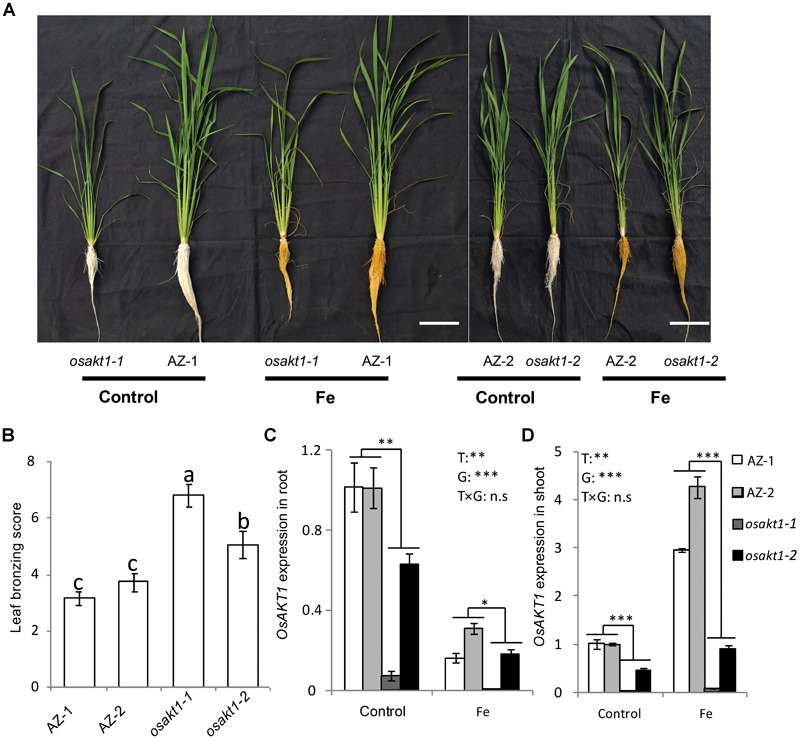
Responses of the *OsAKT1* lines exposed to Fe toxicity (17.9 mM Fe^2+^ for 5 days). **(A)** Photos of *OsAKT1* lines in both control and Fe treatments. **(B)** Leaf bronzing score in Fe treatment. *OsAKT1* expression in the root **(C)** and shoot **(D)**. Vertical bars represent the mean values ± standard errors (*N* = 12 in B, *N* = 3 in C and D). Different letters above the bars indicate the differences were significant at *P* < 0.05 by *post hoc* Tukey’s test. T: treatment; G: genotype; T × G: treatment by genotype interaction; ^∗^ *P* < 0.05; ^∗∗^ *P* < 0.01; ^∗∗∗^ *P* < 0.001, n.s: not significant. Bar scale = 10 cm.

Both AZ and mutant lines showed similar growth after exposure to Fe toxicity (17.9 mM Fe^2+^ for 5 days) without showing reduced shoot or root biomass (Figure 2A and Supplementary Figure S1). However, Fe treatment led to marked leaf bronzing symptoms in both mutant and AZ lines (Figure 2B). Both *osakt1-1* and *osakt1-2* showed higher leaf bronzing score than AZ lines indicating higher sensitivity to Fe toxicity. Relative expression of *OsAKT1* in root and shoot were analyzed with quantitative RT-PCR. Fe treatment significantly suppressed the expression of *OsAKT1* in roots, while the opposite was observed in shoots where *OsAKT1* expression was induced (Figure 2C,D). Both *osakt1-1* and *osakt1-2* showed some residual mRNA expression values which were significantly lower than in AZ-1 and AZ-2, consistent with the semi-quantitative RT-PCR (Figure 1C). Moreover, the knock-down effect in *osakt1-1* was more pronounced than in *osakt1-2*.

### H_2_O_2_ Detection in Leaves

**FIGURE 3:**
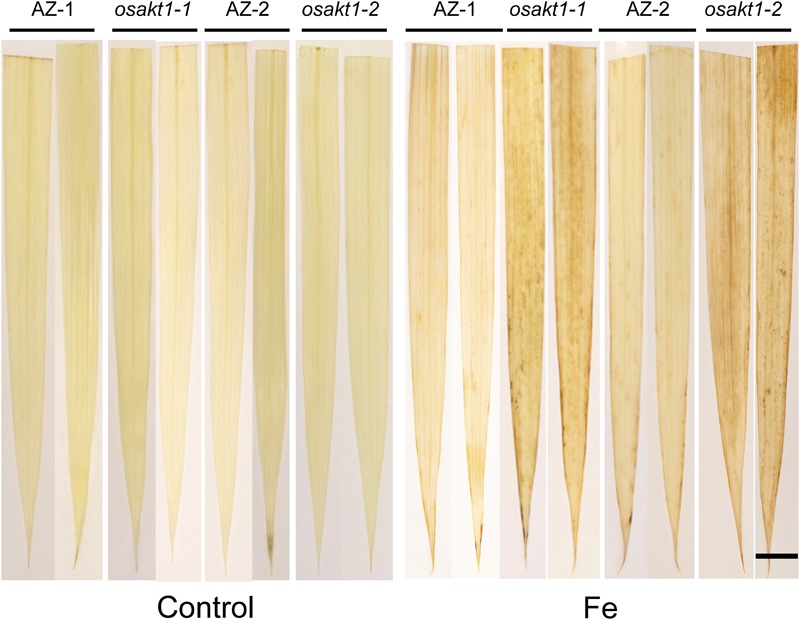
Leaf ROS staining of *OsAKT1* lines in both control and Fe treatment (17.9 mM Fe^2+^ for 5 days). Bar scale = 1 cm. AZ, azygos line.

H_2_O_2_ generation in leaves was visualized by DAB staining (Figure 3). In control conditions, no differences for the H_2_O_2_ generation between mutant lines and AZ lines were observed. Fe treatment markedly induced the H_2_O_2_ production in all the lines, but more so in *osakt1-1* and *osakt1-2* than in AZ-1 and AZ-2.

### Leaf Spectral Reflectance Indices

**FIGURE 4:**
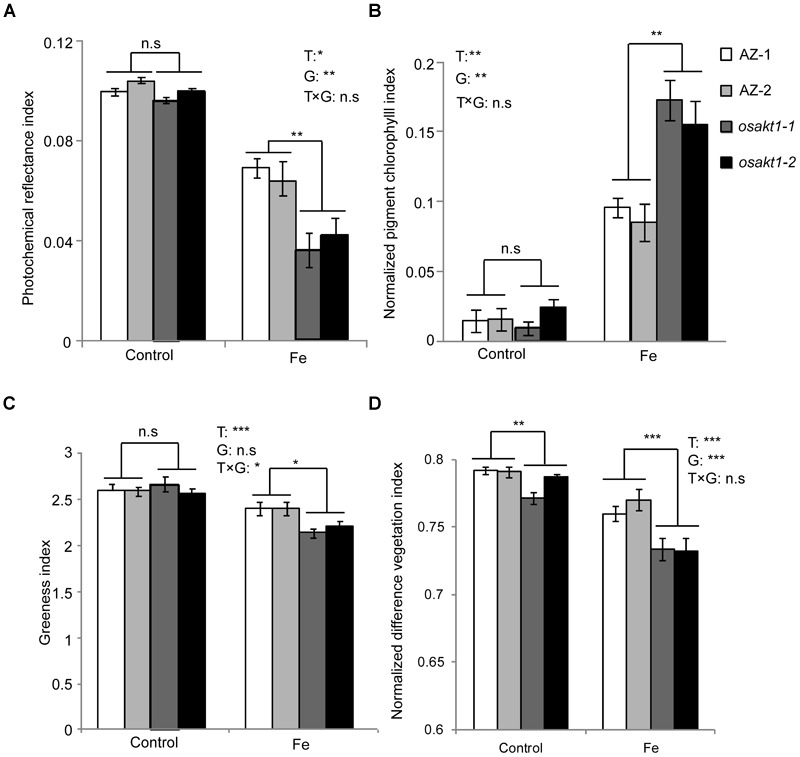
Leaf spectral reflectance indices of *OsAKT1* mutant lines. **(A)** Photochemical reflectance index, **(B)** normalized pigment chlorophyll index, **(C)** greenness index, and **(D)** normalized difference vegetation index. Vertical bars represent mean values ± standard errors (*N* = 6). T: treatment; G: genotype; T × G: treatment by genotype interaction; ^∗^*P* < 0.05; ^∗∗^*P* < 0.01; ^∗∗∗^*P* < 0.001; n.s: not significant; AZ: azygos line.

Leaf spectral reflectance indices were monitored as stress indicators. Fe treatment significantly reduced the PRI indicating that Fe toxicity can severely limit photochemical reactions thus impairing the photosynthesis process. Moreover, mutant lines showed more pronounced decreases than AZ lines indicating that the loss function of *OsAKT1* leads to higher sensitivity to Fe toxicity (Figure 4A). For NPCI, which is inversely related to chlorophyll content, Fe treatment significantly increased the index in both mutant and AZ lines. A significant genotypic difference was only detected in the Fe treatment indicating that mutation of the *OsAKT1* gene led to higher sensitivity to excess Fe (Figure 4B). Based on the results, we conclude that *OsAKT1* is crucial for rice plants to maintain the chlorophyll content subjected to Fe stress. Additionally, leaf GI and NDVI were drastically reduced by Fe treatment. Both AZ-1 and AZ-2 performed significantly better than the mutant lines in Fe treatment (Figure 4C,D). The results indicate the crucial roles of *OsAKT1* in the tolerance to Fe toxicity in rice.

### Gas Exchange and Photosynthetic Activity

**FIGURE 5:**
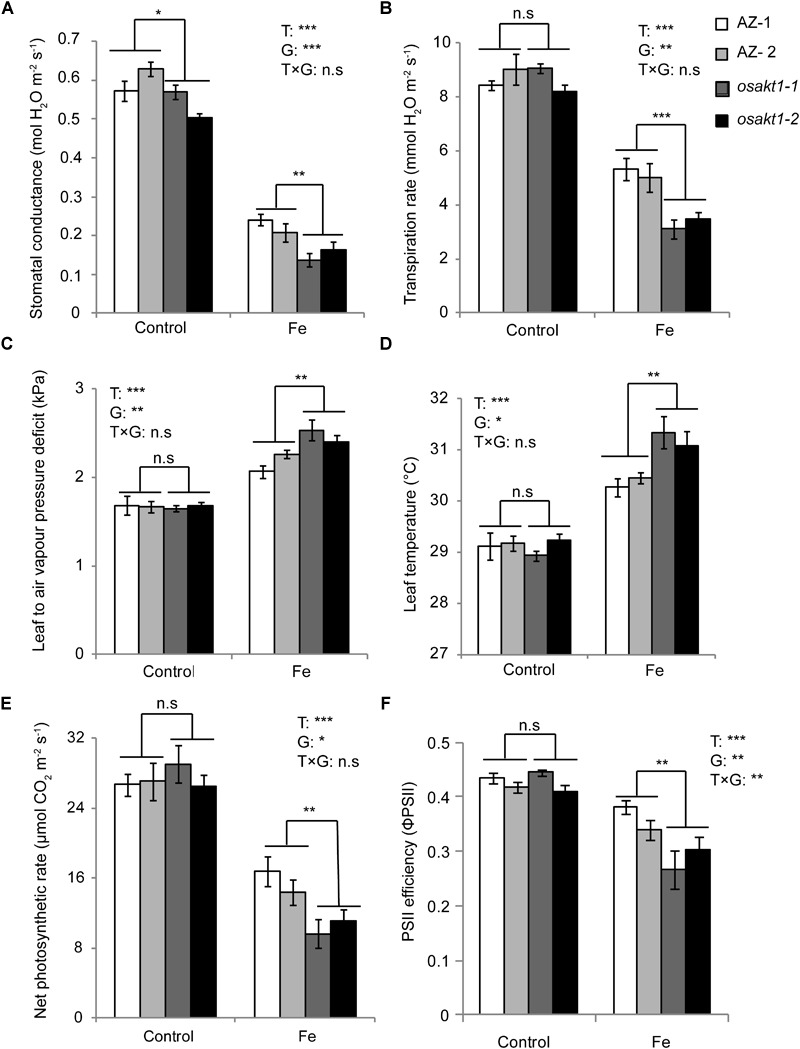
Responses of gas exchange and chlorophyll fluorescence in *OsAKT1* mutant lines exposed to Fe toxicity (17.9 mM Fe^2+^ for 5 days). **(A)** Stomatal conductance, **(B)** transpiration rate, **(C)** leaf to air vapor pressure deficit, **(D)** leaf temperature, **(E)** net photosynthetic rate and **(F)** photosystem II efficiency were measured on the first and second fully expanded leaves of the main tiller. Vertical bars represent mean values ± standard errors (*N* = 6). T: treatment; G: genotype; T × G: treatment by genotype interaction; ^∗^ *P* < 0.05; ^∗∗^ *P* < 0.01; ^∗∗∗^ *P* < 0.001; n.s: not significant; AZ: azygos line.

The impact of Fe toxicity on the photosynthesis was investigated by gas exchange and chlorophyll fluorescence analysis. Stomatal conductance in both AZ and mutants were strongly inhibited by Fe treatment (Figure 5A), and mutants showed significantly lower conductance than AZ lines. Furthermore, leaf transpiration rate was significantly decreased in the Fe treatment. A significant difference was observed between mutant and AZ lines (Figure 5B) in the Fe treatment. Leaf-to-air water pressure deficit together with leaf temperature was induced by Fe toxicity. For both traits, mutant lines showed a higher degree of responses to Fe toxicity (Figure 5C,D).

Regarding net photosynthetic rate (A), photosystem II efficiency (ΦPSII) in both mutant and AZ lines were suppressed by Fe toxicity. In the Fe treatment, *osakt1-1* and *osakt1-2* showed significantly lower net photosynthetic rate than AZ lines (Figure 5E). Moreover, the PSII efficiency in mutant lines was significantly reduced by Fe toxicity compared to AZ lines (Figure 5F). Therefore, the loss-of-function of *OsAKT1* leads to more sensitivity to Fe stress in terms of photosynthetic activities in rice plants.

### Potassium and Fe Measurement in Different Tissues

**FIGURE 6:**
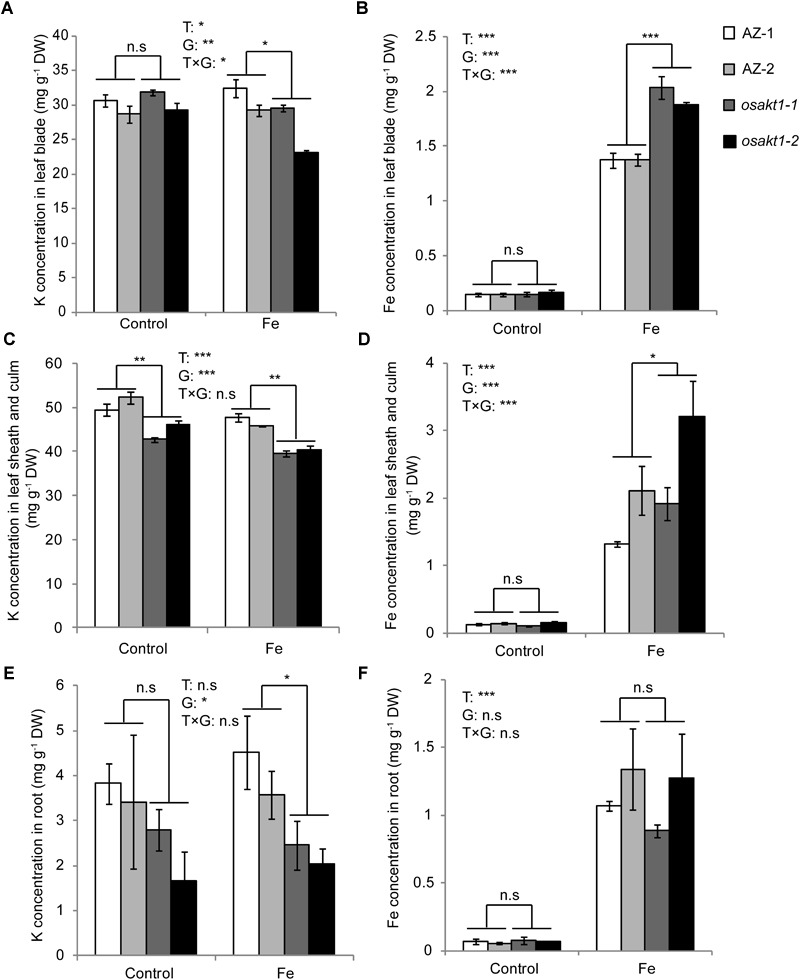
K and Fe concentration in different tissues of *OsAKT1* mutant lines exposed to Fe toxicity (17.9 mM Fe^2+^ for 5 days). **(A)** K concentration in leaf bade, **(B)** Fe concentration in leaf blade, **(C)** K concentration in leaf sheath and culm, **(D)** Fe concentration in leaf sheath and culm, **(E)** K concentration in the root, and **(F)** Fe concentration in the root. Vertical bars represent mean values ± standard errors (*N* = 4). T: treatment, G: genotype, T × G: treatment by genotype interaction. ^∗^ *P* < 0.05; ^∗∗^ *P* < 0.01; ^∗∗∗^ *P* < 0.001; n.s: not significant; AZ: azygos line.

K and Fe concentrations were analyzed in various tissues including leaf blades, SC, and roots. Fe treatment significantly reduced the K concentrations in shoots (Figure 6A,C) but not in the roots (Figure 6E). In the control treatment, *osakt1-1* and *osakt1-2* showed decreased K concentration only in the SC compared to AZ lines (*P* < 0.01, Figure 6C). However, when exposed to excess Fe, the mutant lines showed consistently lower K concentration in all tissues. Fe treatment markedly increased the Fe concentrations in all tissues (*P* < 0.001, Figure 6B,D,F). Moreover, the mutants showed markedly higher Fe concentration in the leaf blade (*P* < 0.001, Figure 6B) and SC (*P* < 0.05, Figure 6D), but not in the root. Thus the mutation of *OsAKT1* leads to reduced K uptake in the Fe treatment, which in turn enhanced Fe translocation from root to shoot, but did not increase Fe concentrations in the root.

### Ionomic Profiling of Rice Plants in Fe Toxicity

**FIGURE 7:**
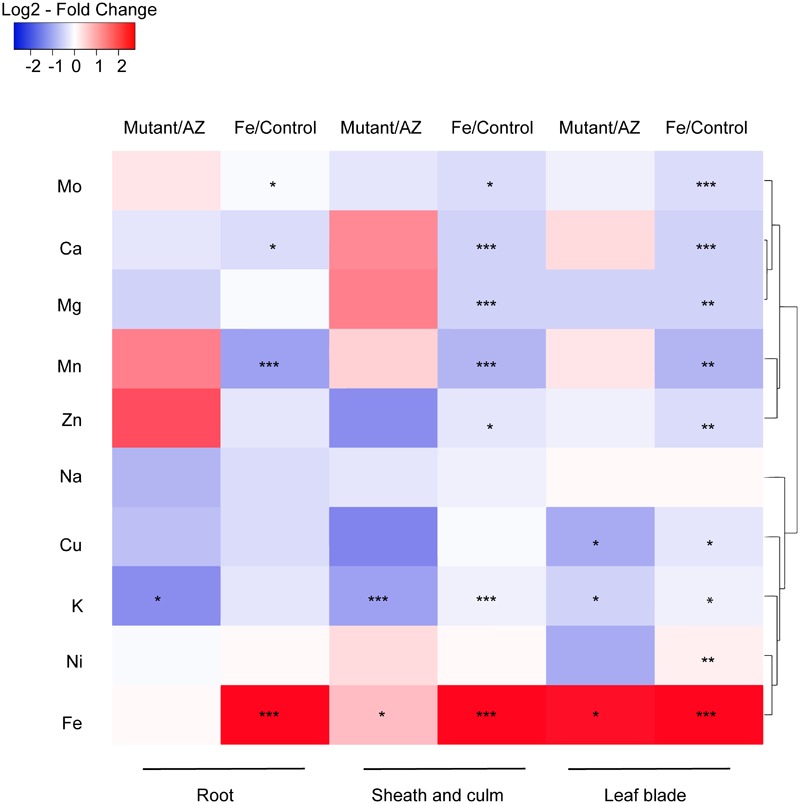
Ionomic profiling of *OsAKT1* lines exposed to Fe toxicity (17.9 mM Fe^2+^ for 5 days). Heatmap was generated using the fold-change value (Log2 transformed) of ions concentrations in various tissues. Blue color indicates the lower element concentration in Fe treatment than control or mutants than azygos lines while red color indicates the opposite results. Only significant differences between treatments or genotypes were shown with asterisks. Hierarchical clustering for the variation patterns of different elements was conducted using the average linkage with the Pearson’s test. AZ: azygos lines; ^∗^ *P* < 0.05; ^∗∗^ *P* < 0.01; ^∗∗∗^ *P* < 0.001.

The impact of excess Fe on other cations uptake in rice was analyzed by ionomic profiling in different tissues including root, SC and leaf blade (Figure 7). In the root, Fe concentration was significantly higher while Mn, Ca and Mo concentrations were lower in the Fe treatment than in control. K concentrations in the mutant lines were significantly lower than in AZ lines (*P* < 0.05), and Fe treatment significantly increased the Fe concentration in SC (*P* < 0.001). Moreover, the concentrations of various elements including Mo, Ca, Mg, Mn, Zn, and K were significantly reduced by Fe treatment. Consistent with root tissue, K concentration in SC was also significantly lower in the mutants than in the AZ lines (*P* < 0.001), whereas Fe concentration was significantly higher in the mutants than in the AZ lines. In the leaf blade, the concentrations of multiple elements including Mo, Ca, Mg, Mn, Zn, Cu, and Ni were reduced by Fe treatment. Fe treatment increased Fe concentrations in leaf blades as expected (*P* < 0.001). The mutant lines showed significantly lower K concentrations in leaf blades, which was associated with reduced Cu but increased Fe concentrations than AZ lines (*P* < 0.05). Clustering analysis was conducted to reveal variation patterns of different cations under Fe toxicity. The variation pattern of Fe and K concentrations showed close linkage, indicating physiological interactions between Fe and K in the rice plants subjected to Fe toxicity.

### Fe Uptake Gene Expression in Root

**FIGURE 8:**
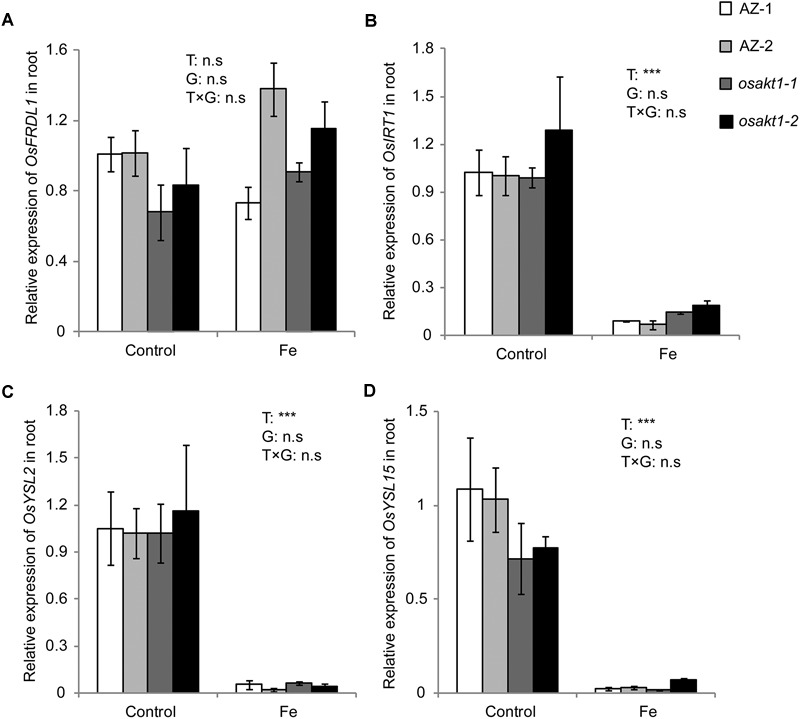
Expression of Fe-transport genes in roots of *OsAKT1* lines exposed to Fe toxicity (17.9 mM Fe^2+^ for 5 days). **(A)** *OsFRDL1*, **(B)** *OsIRT1*, **(C)** *OsYSL2*, and **(D)** *OsYSL15* expression in the root were analyzed with quantitative RT-PCR. Vertical bars represent mean values ± standard errors (*N* = 3). Sequences of the primers used in the test were listed in Supplementary Table S1. T: treatment; G: genotype; T × G: treatment by genotype interaction; ^∗∗∗^ *P* < 0.001, n.s: not significant; AZ: azygos lines.

Due to the differentially regulated shoot Fe concentrations (leaf blade and SC) between mutants and AZ lines (Figure 6), the expression levels of Fe transport genes in root were analyzed to elucidate their possible roles in Fe uptake into the shoot tissues. Four genes including *OsFRDL1, OsIRT1, OsYSL2*, and *OsYSL15* were tested in both control and Fe toxic conditions (Figure 8). Fe treatment significantly reduced the expressions of *OsIRT1, OsYSL2*, and *OsYSL15* in the root. However, no genotypic differences were observed in the four genes expressions, indicating the root to shoot Fe transport genes were not affected by the loss-of-function of *OsAKT1*.

### Fe Concentration in Root Plaque and Xylem Sap

**FIGURE 9:**
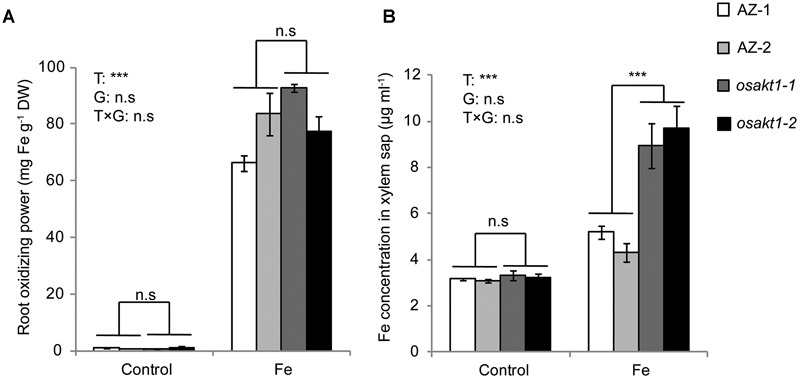
Root oxidizing power and Fe concentration in xylem sap of *OsAKT1* mutant lines exposed to Fe toxicity (17.9 mM Fe^2+^ for 5 days). **(A)** Root oxidizing power. **(B)** Fe concentration in the xylem sap. Vertical bars represent mean values ± standard errors (*N* = 3). T: treatment; G: genotype; T × G: treatment by genotype interaction; ^∗∗∗^ *P* < 0.001; n.s: not significant; AZ: azygos line.

We further investigated the possible mechanisms involved in Fe translocation from root to shoot under Fe toxicity. That lower Fe concentration in the SC in AZ lines was not due to exclusion at the root surface is confirmed by lack of significant differences in root oxidizing power (Figure 9A) between mutants and AZ lines. However, *osakt1-1* and *osakt1-2* showed significantly higher Fe concentration in the xylem sap than AZ lines in the early stage (2 h) of Fe stress (Figure 9B), indicating that root-to-shoot translocation of Fe was affected due to mutation of *OsAKT1*.

## Discussion

In this study, we investigated a potassium ion channel gene *OsAKT1*, which was nominated as a candidate gene underlying an Fe uptake QTL in our previous study (Matthus et al., 2015). In GWAS, *OsAKT1* was identified in a linkage block flanking a highly significant SNP marker on chromosome 1, which was associated with variations of the shoot Fe concentration among 329 Asian rice accessions. By analyzing Tos17 insertional mutants, we confirmed that loss-function of *OsAKT1* in rice led to lower K concentrations in various tissues (Figure 6A,C,E), thereby confirming the findings in previous reports (Fuchs et al., 2005; Obata et al., 2007). Compared to AZ lines, *OsAKT1* mutants showed similar growth after 5 weeks in control conditions despite lower potassium concentration in the SC (Figure 6C). The results were consistent with the findings described by Ahmad et al. (2016), in which, *OsAKT1* mutants showed similar relative growth rate as wildtypes in the absence of stress when rice plants were also grown for 5 weeks. However, Li et al. (2014) observed reduced shoot growth in a T-DNA insertional mutant line for *OsAKT1* compared to the wildtype when both lines were grown only for 1 week. The loss-of-function of *OsAKT1* might be compensated by other K transporters (Ahmad et al., 2016). However, due to the low-capacity transport activities of, e.g., HAK/KUP family transporters (Yang et al., 2014), mutant plants require an extended period to restore growth.

Fe is transported to the shoots via transpiration stream in the xylem (Curie and Briat, 2003). Rice plants responded to excess Fe by decreasing stomatal conductance (Figure 5A), thus lowering the transpiration rate (Figure 5B), which might be considered as an avoidance mechanism to reduce Fe uptake. However, previous studies reported that sensitive genotypes showed a more pronounced decrease in stomatal conductance and transpiration rate than tolerant genotypes (Dufey et al., 2009; Pereira et al., 2013). It suggests that stomata closure is a stress symptom rather than a tolerance mechanism of the rice plants subjected to excess Fe. As a consequence of the lower K uptake, mutant lines showed lower stomatal conductance than AZ lines in control conditions (Figure 5A). In Fe treatment, mutant lines showed reduced stomatal conductance and transpiration rate than AZ lines indicating more sensitivity (Figure 5A,B). Other parameters including ROS generation (Figure 3), chlorophyll content (Figure 4) and photosynthesis efficiency (Figure 5E) also showed the same trend leading to the conclusion that *OsAKT1* is involved in Fe toxicity tolerance in rice.

Apart from inducing oxidative stress in rice plants (Quinet et al., 2012; Höller et al., 2015; Wu et al., 2017), Fe toxicity also affected the uptake of other essential elements into different tissues (Figure 7). The reductions in nutrients uptake might be caused by the damage of root systems (Wu, 2016) or by the root plaque formed through the oxidation and precipitation of Fe (Wu et al., 2012, 2014; Zhou et al., 2015). Additionally, excess Fe can reduce the expression of metal (e.g., Zn, Mn) transporter genes in roots as suggested by previous transcriptome analyses (Quinet et al., 2012; Wu et al., 2017). Thus, Fe toxicity leads to multiple nutrient disorders causing negative feedback on growth and physiological traits. In Fe toxic conditions, K homeostasis plays a vital role in regulating excess Fe translocation into shoot tissues.

Interactions between K and excess Fe were previously reported in *Arabidopsis*, in which the tolerance to excess Fe was found to be positively associated with root K status, which was mediated by ethylene and NO (Li et al., 2015; Zhang et al., 2018). Moreover, the application of K can restore primary root growth in *Arabidopsis* plants subjected to excess Fe (Li et al., 2016). However, contradictory results were found in rice studies regarding the interactions between K and Fe stress (Li et al., 2001). K can mitigate Fe stress in rice when it was applied in the form of K_2_SO_4_ (Yamauchi, 1989; Li et al., 2001), while no protecting effect was observed when K was applied as KCl (Mehraban et al., 2008). In this study, however, we clearly showed that adequate uptake of K could protect rice plants exposed to excess Fe by regulating Fe translocation from root to shoot in Fe toxic conditions (Figure 7).

Long-distance transport of Fe is facilitated by several genes including *OsFRDL1* for Fe translocation to the shoot as a Fe-citrate complex (Yokosho et al., 2009), *OsIRT1* (Ishimaru et al., 2006), *OsYSL2* (Koike et al., 2004), *OsYSL15* (Inoue et al., 2009). The genes responsible for Fe transport were highly induced by Fe deficiency and suppressed by excess Fe (Nozoye et al., 2007; Zheng et al., 2009; Quinet et al., 2012; Wu et al., 2017). In our study, no significant differences were observed for the Fe translocation genes between mutants and AZ lines (Figure 8). These observations suggest that the regular Fe transporters play a less critical role in Fe toxic conditions and other physiological processes caused the differences in shoot Fe concentrations between *OsAKT1* mutants and AZ lines.

**FIGURE 10:**
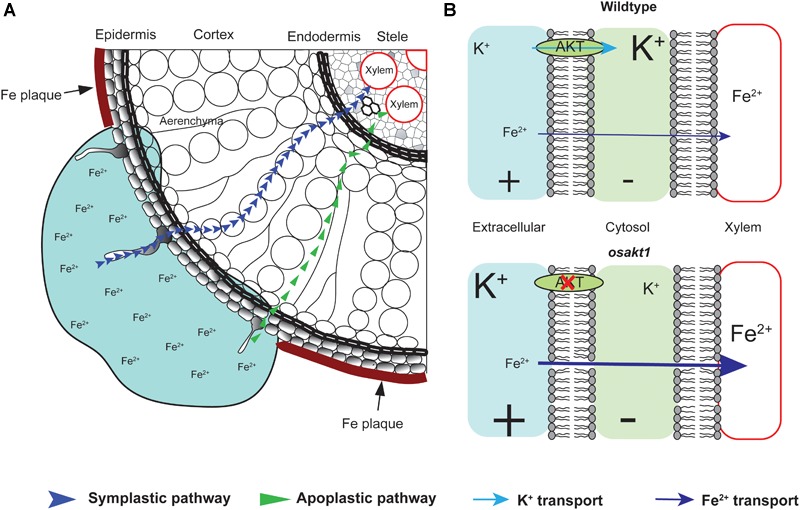
A proposed conceptual model explaining the different xylem Fe concentration observed in *OsAKT1* mutant and wildtype lines. **(A)** In Fe toxic conditions, Fe^2+^ ions enter root cells via both apoplastic and symplastic pathway. Fe plaque formed by root oxidizing power on the root surface did not significantly differ between mutant and wildtype lines. **(B)** Loss-of-function of *OsAKT1* in the mutant lines (lower) leads to lower intracellular K^+^ concentration and thus to a higher degree of hyperpolarization of the plasma membrane than wildtypes (Upper). More hyperpolarized membrane will facilitate more Fe^2+^ ions being loaded into the xylem sap and further transported into the shoots. Different font size of K^+^ and Fe^2+^ indicates different concentration. +, positively charged; -, negatively charged.

Fe plaque on root surface is considered as an essential factor affecting shoot Fe concentration in rice plants exposed to Fe toxic conditions, which depends on the root oxidizing power (Engel et al., 2012; Wu et al., 2014). However, no significant differences between mutants and AZ lines were observed for the amount of Fe oxidized and precipitated on the root surface (Figure 9A), demonstrating that this mechanism did not explain the differences of shoot Fe concentrations. Therefore we explored the xylem as the primary route of Fe transport from root to shoot via the transpiration stream (Morrissey and Guerinot, 2009). Based on our gas exchange measurements (Figure 5B) we can exclude the idea that higher transpiration rate was responsible for Fe accumulation in *OsAKT1* mutants. Instead, higher accumulation of Fe in shoots can be explained with enhanced Fe levels in the xylem sap of *OsAKT1* mutants (Figure 9B), which showed significantly lower root K concentrations than AZ lines (Figure 7). A conceptual model explaining the different level of Fe in the xylem sap between AZ lines and mutant lines was proposed (Figure 10). In Fe toxic conditions, excess Fe^2+^ ions enter the roots via both symplastic and apoplastic pathway (Becker and Asch, 2005). Excess Fe^2+^ ions react with H_2_O_2_ leading to the generation of hydroxyl radicals, which will directly activate outward-rectifying K^+^ channels, e.g., GORK leading to K^+^ leak (Demidchik et al., 2010). Moreover, non-selective cation channels (NSCC) can also be activated by excess Fe^2+^ via NO-mediated pathway leading to K^+^ loss (Zhang et al., 2018). Massive K^+^ leak from the cytosol will hyperpolarize the plasma membrane in root cells (Wang and Wu, 2013). Also due to the loss-of-function of *OsAKT1*, the mutant lines are less capable of restoring cytosolic K^+^ content (Figure 6E) (Li et al., 2014; Ahmad et al., 2016). The plasma membrane in the mutant lines might show a higher degree of hyperpolarization than the AZ lines, which will facilitate more Fe^2+^ uptake in the roots and further transport to the shoots. These mechanisms were likely involved in a higher level of Fe translocation to the xylem in *OsAKT1* mutant lines.

In conclusion, our study demonstrated the importance of endogenous K homeostasis for the shoot accumulation of Fe in rice plants grown in Fe toxic conditions. Targeting the *OsAKT1* gene as an essential hub of K homeostasis in rice may thus be a promising strategy in the breeding of adapted rice varieties.

## Author Contributions

L-BW and MF conceived the project, designed the experiments, analyzed the data, and wrote the manuscript. L-BW, FH, and AW conducted the experiments.

## Conflict of Interest Statement

The authors declare that the research was conducted in the absence of any commercial or financial relationships that could be construed as a potential conflict of interest.

## Footnotes

1 http://tos.nias.affrc.go.jp

2 http://www2.heatmapper.ca/
