# Supplementary material for: Potassium Ion Channel Gene OsAKT1 Affects Iron Translocation in Rice Plants Exposed to Iron Toxicity
Source: Front Plant Sci. 2019 May 8;10:579. doi: 10.3389/fpls.2019.00579 (PMC6517512; doi:10.3389/fpls.2019.00579)
Supplement: Supplementary file 1 [file Image_1.pdf]

Supplementary Figure S1

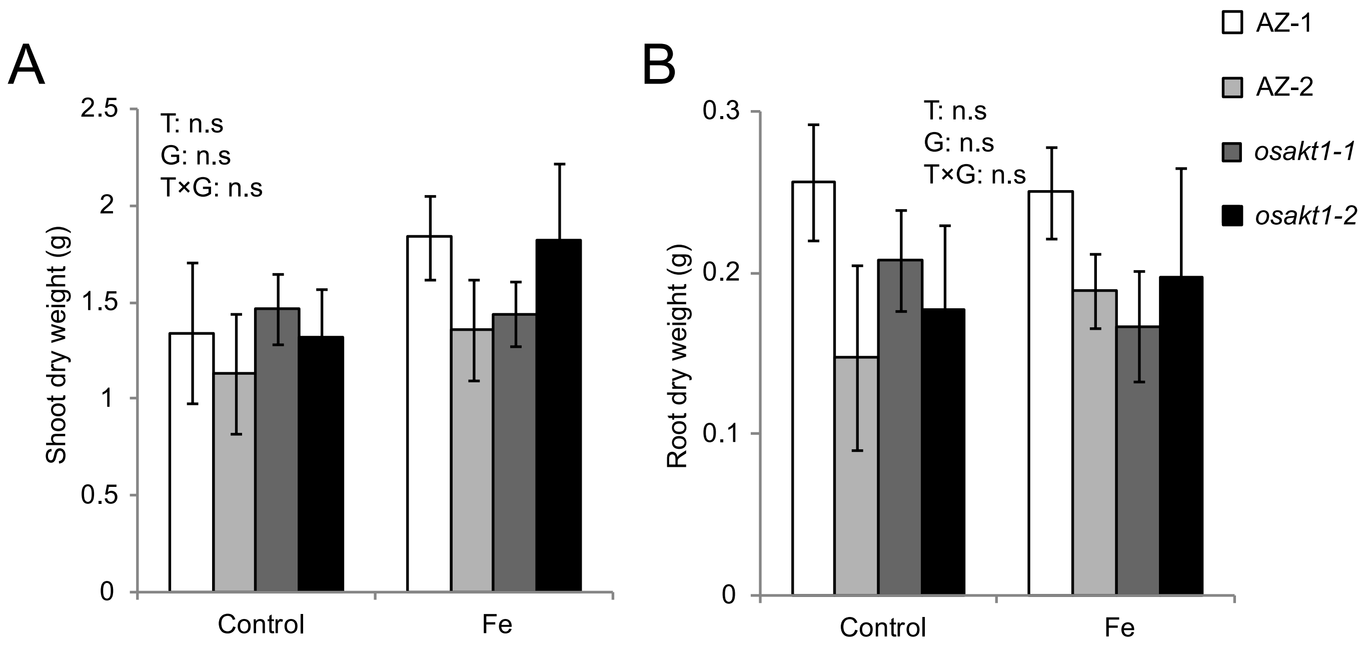

Supporting Information Figure S1. Shoot and root biomass of *OsAKT1* mutant lines exposed to Fe toxicity (1,000 mg Fe^{2+} L^{-1} for 5 days). (A) Shoot dry weight. (B) Root dry weight. Vertical bars represent mean values ± standard errors (N=4). T: treatment; G: genotype; T×G: treatment by genotype interaction; n.s: not significant; AZ: azygous lines,
