# Supplementary material for: Potassium Ion Channel Gene OsAKT1 Affects Iron Translocation in Rice Plants Exposed to Iron Toxicity
Source: Front Plant Sci. 2019 May 8;10:579. doi: 10.3389/fpls.2019.00579 (PMC6517512; doi:10.3389/fpls.2019.00579)
Supplement: Supplementary file 2 [file Table_1.pdf]

Supplementary Table 1. Sequences of the primers used in this study were listed below.

Purpose	Primer_ID	Sequence (5‘-3‘)
NG1928 genotyping	P1_F	CGTGAGGCCATGACATATTG
	P1_R	AATGGCTGGTGTCGTAAAGG
NC2778 genotyping	P2_F	AGCTTGTTGCTGCTTGGATT
	P2_R	TAGGCTAGAGCCACTGCCAT
Tos17 primer	Tos_tail6	AGGTTGCAAGTTAGTTAAGA
qRT-PCR for *OsAKT1*	qAKT1_F	GCTTCCAAAGGAAACGAGCAA
	qAKT1_R	GCAAGCGTATAAGCCCGTGTC
Reference gene, *OsUBQ5*	UBQ5_F	ACCACTTCGACCGCCACTACT
	UBQ5_R	ACGCCTAAGCCTGCTGGTT
qRT-PCR for *OsFRDL1*	qFRDL1_F	TCACCAATGCTAAGGCCTGC
	qFRDL1_R	AACCACGGAAAACACCCTG
qRT-PCR for *OsIRT1*	qIRT1_F	TTCGCCGTCGTCAAGGC
	qIRT1_R	GGCGAGGTGAGGTTGTTGA
qRT-PCR for *OsYSL2*	qYSL2_F	TGCTGAGTTCGACATGGTATG
	qYSL2_R	GGCCGGTCTATCCATTACCT
qRT-PCR for *OsYSL15*	qYSL15_F	ATCTCACCTTGACATCGCCG
	qYSL15_R	AAACGCCCTGTAGAACAGCA

OsAKT1: potassium ion channel, LOC_Os01g45990; OsUBQ5: rice ubiquitin gene, LOC_Os01g22490, *OsFRDL1*: FRD3-like protein 1, LOC_Os03g11734, *OsIRT1*: iron-regulated transporter 1, LOC_Os03g46470, *OsYSL2*: yellow strip-like gene 2, LOC_Os02g43370, *OsYSL15*: yellow strip-like gene 15, LOC_Os02g43410. qRT-PCR: quantitative reverse transcription PCR, F: forward, R: reverse.
